# Trends in intentional and unintentional poisonings among older adults - A national register-based study in Sweden

**DOI:** 10.1186/s12877-023-03973-4

**Published:** 2023-05-15

**Authors:** J. Möller, E. Lindholm, P. Fredlund, M. Vaez, Y. Liang, L. Laflamme

**Affiliations:** 1 Department of Global Public Health, Karolinska Institutet, K9 Global Folkhälsa, K9 GH, 171 77 Stockholm, Sweden; 2 Department of Living Conditions and Lifestyles, The Public Health Agency of Sweden, Stockholm, Sweden; 3 Department of Clinical Neuroscience, Division of Insurance Medicine, Karolinska Institutet, Stockholm, Sweden

**Keywords:** Poisonings, Trends, Older adults, Register-based

## Abstract

**Background:**

Among older people intentional poisoning outnumber unintentional ones. While there are indications that time trends differ by poisoning intent, studies are scarce. We assessed how the annual prevalence of intentional and unintentional poisoning changed over time, overall and by demographic groups.

**Methods:**

We conducted a national open cohort study of individuals aged 50–100 years, resident in Sweden during 2005–2016. Individuals were followed up in population-based registers for their demographic and health attributes from 2006–2016. Annual prevalence of hospitalization and death by poisoning intent (unintentional vs. intentional or undetermined; ICD-10 definitions) were compiled for the categories of four demographic attributes (age, sex, marital status, and birth cohort “baby boomers”). The time trends were assessed by multinomial logistic regression with year as an independent variable.

**Results:**

The annual overall prevalence of hospitalization and death by intentional poisonings consistently exceeded that of unintentional poisonings. There was a significant downward trend in intentional poisonings but not in unintentional ones. This difference in trends also applied when considering men and women separately, married and unmarried people, the young-old individuals (but not the older- or oldest-old ones), and the baby boomers and non-baby boomers. The largest demographic differences within intent were found between married and unmarried people, and the smallest one between men and women.

**Conclusion:**

As expected, the annual prevalence of intentional poisonings considerably exceed that of unintentional ones among Swedish older people. The recent trends reveal a significant reduction of intentional poisonings, consistent across a range of demographic attributes. The scope for action regarding this preventable cause of mortality and morbidity remains considerable.

**Supplementary Information:**

The online version contains supplementary material available at 10.1186/s12877-023-03973-4.

## Introduction

Older people are a vulnerable group for poisoning [1, 2] and as this population is on the increase globally, this preventable cause of morbidity and mortality also rises. Older people are more vulnerable to poisoning because they are more exposed to toxic agents [1, 3–5] and because of how they respond to those substances, physiologically and pathologically [6, 7]. Acknowledged risk factors include the impairment of a range of physiological systems characterized by a reduction in functional reserves,an increased incidence of subclinical and clinical diseases (degenerative, malignant, and infection-related) paralleled with an increase in the consumption of medications; an increased risk of adverse drug reactions and medication errors; and an individual vulnerability linked to impoverished social and economic context [1, 4, 6, 7].

Further, poisoning is one of the methods by which older people attempt to end their lives, afflicted by health-related issues (e.g., psychiatric illness, functional impairment, poor physical health) or social factors, circumstantial (like stressful life events) or more long-lasting, like poor social networks [8]. In Europe, self-poisoning is in fact the most common method of deliberate-self harm among older people [9, 10] and drugs is by far the most common poisoning agent of those poisonings [11, 12], as it is for unintentional poisonings [13, 14]. In Sweden, the context of the present study, as in other countries [2, 11], intentional poisonings are far more common than unintentional ones among older adults [15] and earlier estimates suggest that the proportion of unintentional poisonings increases in later life [16]. This is also reflected in a recent study of consultations to the Dutch Poisons Information Center showing a substantial increase in the number and proportion of patients aged 65 years and above between 2010 and 2020 [14].

In recent years, to the best of our knowledge, studies on poisoning trends among older people over time are surprisingly scarce, except for a few on fatal poisonings: one on accidental poisoning in Pennsylvania, USA [13] and one on fatal drug poisoning from Spain [11]. During the period 2000–2018, the Spanish study shows an increase in both suicidal poisonings and accidental drug poisonings among individuals aged 65 years and above, with an average change of 16.2% and 7.7% respectively. An increase in accidental poisoning mortality, all causes aggregated, was also observed in Pennsylvania, from 1979 to 2014. Overall, the rate ratios increased more than 14-fold.

In Sweden, for all ages aggregated reports for the period 1997–2013 show a decrease in hospitalization for intentional poisonings and a marked increase in unintentional ones [15]. Whether this applied to older people has not been investigated and recent follow ups are lacking. A better insight on trends in poisoning among older people is warranted due to the prediction of a rise in intentional poisonings attributable to the increasing number of older people in the total population [17] and to the increasing proportion of ‘baby boomers’ (born 1946–1964) among them, a group with a particularly high propensity for suicide [8].

In this study, we assessed whether there are significant changes over time in the annual prevalence of hospitalization and death by intentional and unintentional poisoning among older people in Sweden, overall and by demographic groups, including birth cohorts of “baby boomers” compared with their counterpart.

## Method

### Design

We conducted a population-based longitudinal study.

### Material

The study population consists of an open cohort of individuals born in 1965 or earlier who have been registered as residents in Sweden at some point in time between 2005 and 2016 in the Swedish Register of the Total Population. At the beginning of the follow-up period the population included 3 512 142 individuals and, in the end (2016), 3 797 469 individuals with an average of 3 689 910 during the study period (see Table S1 for annual distribution of population included); 50 years was the age of inclusion and 100 years the latest age of exclusion. Individuals also excluded were those who died or emigrated in each corresponding year. Individuals immigrating back were included again from the year they did so. All included individuals were followed in population-based registers for their demographic and health attributes between 2006–2016, including the Register of the Total Population, the Longitudinal Integration Database for Health Insurance and Labor Market Studies, both held by Statistics Sweden, and the National Patient Register and the Cause of Death Register, held by The National Board of Health and Welfare.

For each year, we considered all first cases of poisoning leading to hospitalization (at least one night) or death. We defined and classified those poisonings by intent: intentional and unintentional as per ICD-10 external causes (e-codes). Intentional poisonings included those classified as intentional (ICD-10 codes: X60-X69) and those, less frequent, of an undetermined cause (ICD-10 codes: Y10-Y19; about 17% of the total material, annual prevalence presented in Figure S1). This was done in accordance with recommendations from the National Centre for Research on Suicide and Prevention of Mental Ill-Health, as a means to avoid underestimation since, in Swedish registers, where the proportion undetermined is relatively high ( all ages aggregated the proportion of undetermined to intentional suicides is about 20%). After psychological assessments a majority of those coded as undetermined intent (70–75%) are considered as suicides [18]. In the remainder of the text those are called intentional poisonings. Unintentional poisonings were classified as the ICD-10 codes X40-X49.

Further, we considered the following demographic characteristics: sex, marital status (married and unmarried), age group (50–64, 65–79, and 80–100 years), and birth cohorts of “baby boomers” (i.e., born 1946–64; yes/no).

### Statistical analysis

To estimate the trends in poisonings the annual prevalence per 100 000 persons was calculated by compiling first incident poisonings over population at risk each year. Population at risk was calculated with each person in the cohort contributing to one year annually during each year of inclusion. The annual prevalence of hospitalization or death due to unintentional and intentional poisonings was compiled overall and by demographic characteristics for the period January 1st 2006 to December 31st 2016; see supplementary tables for the annual frequency and distribution of the population by demographic characteristic for intentional poisonings (Table S2) and unintentional poisoning (Table S3). We also compiled annual male-to-female ratios and unmarried-to-married ratios.

Multinomial logistic regression was used to assess the trend in prevalence of both intentional and unintentional poisonings over time, with year as an independent variable in the model, and present *p*-values (P) for statistical significance in trend. The linear regression was performed for assessing the trend of ratio (e.g., male to female ratio, and unmarried to married ratio) across years.

For data analysis, we used SAS version 9.4 and, for testing the significance of the trends, IBM SPSS 28 for Windows (IBM SPSS Inc., Chicago, Illinois, USA).

### Results

During the study period, a total of 24 964 intentional and 12 149 unintentional poisonings leading to hospitalization or death occurred, the distribution of which by demographic characteristics is presented in Table 1.

**Table 1:** Distribution of poisonings leading to hospitalization and death by intent and demographic characteristics in Sweden (2006–2016)

Characteristics	Intentional *n* = 24 964		Unintentional *n* = 12 149	
	n	%	n	%
Sex				
Men	11 053	44.3	6187	50.9
Women	13 911	55.7	5962	49.1
Age (in years)				
50—64	15 909	63.2	4764	39.2
65—79	6260	25.0	3981	32.8
80—100	2795	11.2	3404	28.0
Marital status				
Married	7753	31.1	3881	32.0
Unmarried	17 156	68.9	8243	68.0
Baby boomer				
Yes	15 729	65.6	4920	43.6
No	8247	34.4	6355	56.4

**Fig. 1:**
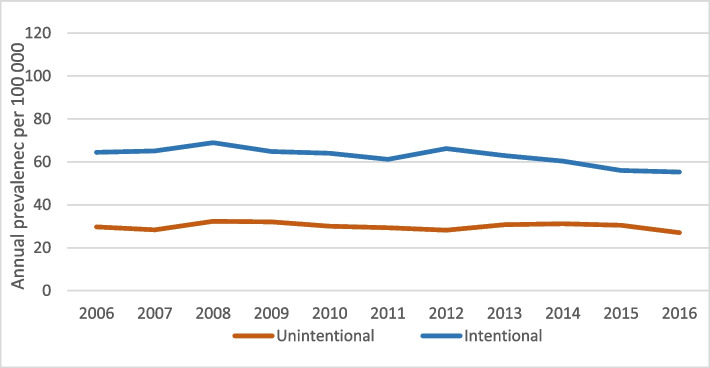
Annual prevalence (per 100,000) of intentional and unintentional poisonings leading to hospitalization or death among older adults in the older adult population (50–100 years), 2006–2016

Figure 1 presents the overall annual prevalence of poisonings leading to hospitalization and death between the years 2006 and 2016 by intent. For any given year, the prevalence of intentional poisonings exceeds that of unintentional ones. Yet, there is a significant downward trend in the former (P for trend < 0.001) but not in the latter (P for trend = 0.168).

**Fig. 2:**
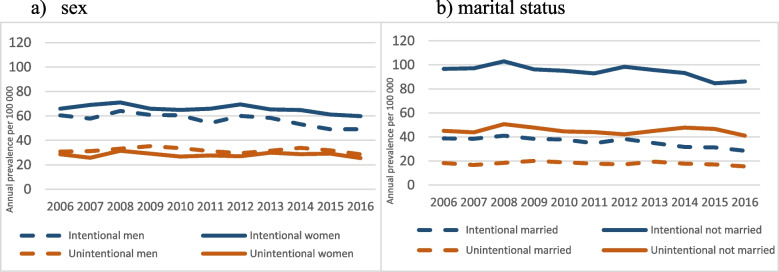
Annual prevalence (per 100 000) of poisonings by intent, stratified by sex (**a**) and marital status (**b**), 2006–2016

Trends by sex and marital status are presented in Figs. 2a and b respectively. The sex-specific annual prevalence is also higher for intentional than unintentional poisoning among both men and women, with significant downwards trends for intentional poisoning in both groups (*p* < 0.001) but not significant for unintentional poisonings (*P* = 0.182 for men and *P* = 0.497 for women). Throughout the study period the annual prevalence of intentional poisoning is consistently higher among women than men and unintentional higher among men than women. Also, as shown in Table 1, the male to female ratio for intentional poisoning decreased over time (P for trend = 0.032; highest in 2006 – 0.92 and lowest in 2015 – 0.80; see supplementary Table S2).

For marital status (Fig. 2b), the annual prevalence of both intentional and unintentional poisoning is higher among those unmarried than their married counterparts, with remarkable differences for intentional poisonings. Here too there are significant downward trends for intentional poisonings for both groups (*P* < 0.001) but not for unintentional poisonings (*P* = 0.084 for married and *P* = 0.146 for unmarried). It is of note that for intentional poisoning there is a significant increase in the unmarried-to-married prevalence ratio over time (see Table 2; P for trend < 0.001; highest in 2016 – 3.02 and lowest in 2006 – 2.50).

**Table 2:** Male to female and unmarried to married annual prevalence ratios of poisoning by intent, 2006–2016

Year	Male to female ratio		Unmarried to married ratio	
	Intentional	Unintentional	Intentional	Unintentional
2006	0.92	1.08	2.50	2.47
2007	0.84	1.21	2.52	2.63
2008	0.90	1.06	2.51	2.75
2009	0.92	1.21	2.50	2.38
2010	0.93	1.25	2.51	2.37
2011	0.82	1.13	2.67	2.47
2012	0.86	1.09	2.57	2.45
2013	0.89	1.05	2.74	2.32
2014	0.82	1.18	2.94	2.69
2015	0.80	1.09	2.71	2.73
2016	0.82	1.12	3.02	2.65
*P-value for trend*	*0.032*	*0.604*	< *0.001*	*0.538*

**Fig. 3:**
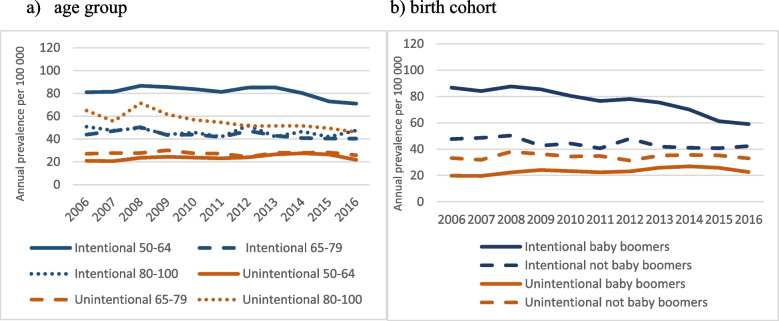
Annual prevalence (per 100 000) of poisonings by intent, age group (**a**) and birth cohort (**b**), 2006–2016

Figure 3 presents the breakdown of time trend by age group (Fig. 3a) and birth cohort (Fig. 3b). Whereas the highest annual prevalence of intentional poisoning is consistently found among people aged 50–64 years, that of unintentional poisoning is among people aged 80–100 years. Yet, time trends for intentional poisoning are significant for the two younger age groups (50–64 and 65–79; *P* < 0.001) but not for the older one (80–100 years; *P* = 0.135) and for both baby boomers and non-baby boomers (*P* < 0.001). For unintentional poisoning the time trend is significant for the youngest and eldest age groups (*P* < 0.001) but not the intermediary one (P for trend = 0.455). Unintentional poisonings have a slight – and significant – tendency to increase over time (P for trend < 0.001) among baby boomers but not among their counterparts (P for trend = 0.919).

## Discussion

### Main findings

This study shows large differences in the annual prevalence of intentional and unintentional poisonings among older adults, overall and between demographic groups. It reveals that in recent years there has been a significant downward trend in intentional poisoning leading to hospitalization and death, but not unintentional ones. Similar results were observed among men and women and married and unmarried people. Age-related results are mixed, with a significant downward time trends for unintentional poisoning among the youngest older people (but not the two other age groups) and among the baby boomers. For intentional poisoning, a similar significant downward trend was found among people aged 80–100 years and among both baby boomers and non-baby boomers. It should be underlined though that the relative steady annual prevalence of unintentional poisoning means that the actual number of those poisonings is on the increase, given that the population at risk increases.

Interestingly, these age-related trends contrast with what would have been expected from earlier national ones for all ages aggregated, for the period 1997–2013, when a decrease in intentional poisonings and a marked increase in unintentional ones were apparent (MSB 2014). Our results contrast even with those obtained in Spain where older people were considered but with a focus on drug-related and fatal poisonings [11]. In that study, from 2000–2018 the increase in intentional poisonings was among people aged 65 years and over and it was even sharper than that among their younger counterparts. Likewise, accidental fatal drug poisoning increased among older adults, but not in the younger group.

Our trends by demographic groups, not reported in earlier studies, indicate that the overall trends are largely replicated in the sub-group analyses. The significant differences between men and women were somehow expectable [19], not least because the majority of the poisonings included in the analyses, albeit severe enough to require hospitalisaion, were non-fatal. This should not be interpreted as an indication that older men in Sweden do not commit deliberate self-harm. Rather, it is known that men in general are inclined to favor methods that are more violent, and they are also more likely to complete suicide. However, some studies indicate that, after the age of 70 years, the rates of deliberate self-harm among men and women begin to converge [9, 10].

Our results are also consistent with previous ones showing a significant protective effect of being married [19]. Nonetheless, it should be underlined that this effect can mask, at least in part, an effect of gender given that women often outlive their partner/husband.

When it comes to age, in our data the absence of remarkable differences in intentional poisonings between the two older age groups (65–79 and 80–100 years) could also be expected, as could those groups’ considerably lower annual prevalence compared to their younger counterparts 50–64 years [9, 19]. It is possible though that a difference would be observable between people from the older age groups (65–79 and 80–100 years) should we have considered completed suicides only [9, 19].

Finally, for the baby boomers our results show both consistent higher levels of intentional poisoning among them compared to their counterparts and a narrowing difference during the study period. This is not exactly in line with the prediction of others, but those predictions were more based on completed suicide, which, as indicated above is only a fraction of the outcome studied here. There might also be changes in the future as the whole population of baby boomers is considered. Not all of them are included in our data as the follow up period ends in 2016.

From another viewpoint, the data at hand are silent about what has happened during the COVID-19 pandemic, a period not included in our data but one that might have affected intentional poisoning as it influenced self-harm in general [20]. Some have indicated, though, that the effects in older populations might have been mixed: protective among those who benefited from strengthened (albeit small) social networks and aggravating among others who did not, for example those living alone [21].

### Strengths and limitations

Major strengths of this study rest on its national coverage, relatively long in time, and the inclusion of demographic data that enabled disaggregated assessments. Although the national registers used are considered, in the main, of high quality, they remain silent as regards poisonings that do not lead to hospitalizations. This, in turn, inevitably leads to an underestimation of the true burden of unintentional and intentional poisoning and can also mask differences between demographic groups, notably age- and sex-related ones.

Registers of the like also have the drawback to lack precision of the actual substance – or combination of substances – responsible for the poisoning. Some have underlined not only the likely difference in the clinical course associated with poisoning by different toxic agents especially among older people [22], and also potential changes in the substances leading to poisoning over time [23].

Given the vulnerability of older people to poisoning and to sustaining severe consequences from poisoning, future studies could help provide greater insight into how substances at risk change over time and whether they differentially impact people from different age groups. Studying individuals who re-injure themselves over time will also be important as this phenomenon can be expected [10, 19].

## Conclusion

As expected, the prevalence of intentional poisonings considerably exceeds that of unintentional ones among older people in Sweden. Although recent trends point to a significant reduction of intentional poisonings, consistent across a range of demographic attributes, there remains considerable scope for improvement in this preventable cause of mortality and morbidity.

## Supplementary Information

**Additional file 1:** **Figure S1.** Graphical presentation of annual prevalence of hospitalized poisonings in Sweden 2006-2016.

**Additional file 2:** **Table S1. **Number of population at risk for intentional and unintentional poisonings in Sweden 2006-2016.

**Additional file 3:** **Table S2.** Annual frequency and distribution of the population across demographic characteristics for intentional poisonings.

**Additional file 4:** **Table S3.** Annual frequency and distribution of the population across demographic characteristics for unintentional poisonings.

## Data Availability

The datasets used and/or analyzed during the current study are available from the corresponding author on reasonable request.
