# Supplementary material for: Trends in intentional and unintentional poisonings among older adults - A national register-based study in Sweden
Source: BMC Geriatr. 2023 May 15;23:296. doi: 10.1186/s12877-023-03973-4 (PMC10184059; doi:10.1186/s12877-023-03973-4)
Supplement: Supplementary file 1 — Additional file 1: Figure S1. Graphical presentation of annual prevalence of hospitalized poisonings in Sweden 2006-2016. [file 12877_2023_3973_MOESM1_ESM.docx]

**Figure S1.** Graphical presentation of annual prevalence of hospitalized poisonings in Sweden 2006-2016.
