# Supplementary material for: Trends in intentional and unintentional poisonings among older adults - A national register-based study in Sweden
Source: BMC Geriatr. 2023 May 15;23:296. doi: 10.1186/s12877-023-03973-4 (PMC10184059; doi:10.1186/s12877-023-03973-4)
Supplement: Supplementary file 2 — Additional file 2: Table S1. Number of population at risk for intentional and unintentional poisonings in Sweden 2006-2016. [file 12877_2023_3973_MOESM2_ESM.docx]

**Table S1.** Number of population at risk for intentional and unintentional poisonings in Sweden 2006-2016.

| **Year** | **Population at risk** |
| --- | --- |
| 2006 | 3 512 142 |
| 2007 | 3 547 076 |
| 2008 | 3 580 202 |
| 2009 | 3 613 768 |
| 2010 | 3 648 710 |
| 2011 | 3 684 436 |
| 2012 | 3 725 012 |
| 2013 | 3 769 868 |
| 2014 | 3 826 090 |
| 2015 | 3 884 208 |
| 2016 | 3 797 469 |
| Average | 3 689 910 |
