# Supplementary material for: Trends in intentional and unintentional poisonings among older adults - A national register-based study in Sweden
Source: BMC Geriatr. 2023 May 15;23:296. doi: 10.1186/s12877-023-03973-4 (PMC10184059; doi:10.1186/s12877-023-03973-4)
Supplement: Supplementary file 3 — Additional file 3: Table S2. Annual frequency and distribution of the population across demographic characteristics for intentional poisonings. [file 12877_2023_3973_MOESM3_ESM.docx]

**Table S2.** Annual frequency and distribution of the population across demographic characteristics for intentional poisonings

|  | 2006 | 2007 | 2008 | 2009 | 2010 | 2011 | 2012 | 2013 | 2014 | 2015 | 2016 | Total |
| --- | --- | --- | --- | --- | --- | --- | --- | --- | --- | --- | --- | --- |
|  | n(%) | n(%) | n(%) | n(%) | n(%) | n(%) | n(%) | n(%) | n(%) | n(%) | n(%) | N |
| **Sex** |  |  |  |  |  |  |  |  |  |  |  |  |
| Men | 1004 (9,1) | 971 (8,8) | 1090 (9,9) | 1047 (9,5) | 1054 (9,5) | 955 (8,6) | 1071 (9,7) | 1058 (9,6) | 981 (8,9) | 921 (8,3) | 901 (8,2) | 11053 |
| Women | 1220 (8,8) | 1289  (9,3) | 1335  (9,6) | 1248 (9,0) | 1239  (8,9) | 1267 (9,1) | 1347 (9,7) | 1279 (9,2) | 1286 (9,2) | 1227 (8,8) | 1174 (8,4) | 13911 |
| **Age** |  |  |  |  |  |  |  |  |  |  |  |  |
| 50 - 64 | 1462 (9,2) | 1467 (9,2) | 1567 (9,8) | 1527 (9,6) | 1486 (9,3) | 1448 (9,1) | 1494 (9,4) | 1504 (9,5) | 1431 (9,0) | 1318 (8,3) | 1205 (7,6) | 15909 |
| 65 - 79 | 487 (7,8) | 535 (8,5) | 586 (9,4) | 528 (8,4) | 555 (8,9) | 543 (8,7) | 639 (10,2) | 602 (9,6) | 584 (9,3) | 597 (9,5) | 604 (9,6) | 6260 |
| 80 - 100 | 275 (9,8) | 258 (9,2) | 272 (9,7) | 240 (8,6) | 252 (9,0) | 231 (8,3) | 258 (10,2) | 231 (8,3) | 252 (9,0) | 233 (8,3) | 266 (9,5) | 2795 |
| **Marital status** |  |  |  |  |  |  |  |  |  |  |  |  |
| Married | 750 (9,7) | 748 (9,6) | 800 (10,3) | 752 (9,7) | 743 (9,6) | 684 (8,8) | 757 (9,8) | 693 (8,9) | 634 (8,2) | 631 (8,1) | 561 (7,2) | 7753 |
| Not married | 1473 (8,6) | 1506 (8,8) | 1620 (9,4) | 1538 (9,0) | 1545 (9,0) | 1533 (8,9) | 1653 (9,6) | 1636 (9,5) | 1630 (9,5) | 1512 (8,8) | 1510 (8,8) | 17156 |
| **Baby boomer** |  |  |  |  |  |  |  |  |  |  |  |  |
| Yes | 1171 (7,4) | 1235 (7,9) | 1387 (8,8) | 1448 (9,2) | 1456 (9,3) | 1472 (9,4) | 1590 (10,1) | 1631 (10,4) | 1608 (10,2) | 1395 (8,9) | 1336 (8,5) | 15729 |
| No | 988 (12,0) | 966 (11,7) | 960 (11,6) | 780 (9,5) | 776 (9,4) | 679 (8,2) | 763 (9,3) | 636 (7,7) | 592 (7,2) | 558 (6,8) | 549 (6,7) | 8247 |
