# Supplementary material for: Trends in intentional and unintentional poisonings among older adults - A national register-based study in Sweden
Source: BMC Geriatr. 2023 May 15;23:296. doi: 10.1186/s12877-023-03973-4 (PMC10184059; doi:10.1186/s12877-023-03973-4)
Supplement: Supplementary file 4 — Additional file 4: Table S3. Annual frequency and distribution of the population across demographic characteristics for unintentional poisonings. [file 12877_2023_3973_MOESM4_ESM.docx]

| **Table S3.** Annual frequency and distribution of the population across demographic characteristics for unintentional poisonings   \|  \| 2006 \| 2007 \| 2008 \| 2009 \| 2010 \| 2011 \| 2012 \| 2013 \| 2014 \| 2015 \| 2016 \| Total \| \| --- \| --- \| --- \| --- \| --- \| --- \| --- \| --- \| --- \| --- \| --- \| --- \| --- \| \|  \| n(%) \| n(%) \| n(%) \| n(%) \| n(%) \| n(%) \| n(%) \| n(%) \| n(%) \| n(%) \| n(%) \| N \| \|  \|  \|  \|  \|  \|  \|  \|  \|  \|  \|  \|  \|  \| | | | | | | | | | | | | | |
| --- | --- | --- | --- | --- | --- | --- | --- | --- | --- | --- | --- | --- | --- | --- | --- | --- | --- | --- | --- | --- | --- | --- | --- | --- | --- | --- | --- | --- | --- | --- | --- | --- | --- | --- | --- | --- | --- | --- | --- | --- | --- | --- | --- | --- | --- | --- | --- | --- | --- | --- | --- | --- |
| **Sex** |  |  |  |  |  |  |  |  |  |  |  |  |  |
| Men | 512 (8,3) | 524 (8,5) | 565 (9,1) | 607 (9,8) | 585 (9,5) | 549 (8,9) | 527 (8,5) | 571 (9,2) | 624 (10,5) | 598 (9,7) | 525 (8,5) | 6187 |  |
| Women | 531 (8,9) | 481 (8,1) | 592 (9,9) | 551 (9,2) | 511 (8,6) | 531 (8,9) | 523 (8,8) | 587 (9,8) | 569 (9,5) | 585 (9,8) | 501 (8,4) | 5962 |  |
| **Age** |  |  |  |  |  |  |  |  |  |  |  |  |  |
| 50 - 64 | 385 (8,1) | 382 (8,0) | 435 (9,1) | 448 (9,4) | 432 (9,1) | 416 (8,7) | 431 (9,0) | 477 (10,0) | 499 (10,5) | 485 (10,2) | 374 (7,9) | 4764 |  |
| 65 - 79 | 304 (7,6) | 319 (8,0) | 329 (8,3) | 371 (9,3) | 350 (8,8) | 361 (9,1) | 333 (8,4) | 395 (9,9) | 407 (10,2) | 421 (10,6) | 391 (9,8) | 3981 |  |
| 80 - 100 | 354 (10,4) | 304 (8,9) | 393 (11,5) | 339 (10,0) | 314 (9,2) | 303 (8,9) | 286 (8,4) | 286 (8,4) | 287 (8,4) | 277- (8,1) | 261 (7,7) | 3404 |  |
| **Marital status** |  |  |  |  |  |  |  |  |  |  |  |  |  |
| Married | 354 (9,1) | 324 (8,3) | 359 (9,3) | 393 (10,1) | 369 (9,5) | 350 (9,0) | 340 (8,8) | 386 (9,9) | 355 (9,1) | 346 (8,9) | 305 (7,9) | 3881 |  |
| Not married | 687 (8,3) | 679 (8,2) | 797 (9,7) | 763 (9,3) | 724 (8,8) | 726 (8,8) | 708 (8,6) | 771 (9,4) | 835 (10,1) | 833 (10,1) | 720 (8,7) | 8243 |  |
| **Baby boomer** |  |  |  |  |  |  |  |  |  |  |  |  |  |
| Yes | 268 (5,4) | 288 (5,9) | 353 (7,2) | 409 (8,3) | 422 (8,6) | 430 (8,7) | 472 (9,6) | 560 (11,4) | 616 (12,5) | 589 (12,0) | 513 (10,4) | 4920 |  |
| No | 689 (10,8) | 634 (10,0) | 726 (11,4) | 664 (10,4) | 603 (9,5) | 582 (9,2) | 499 (7,9) | 533 (8,4) | 514 (8,1) | 483 (7,6) | 428 (6,3) | 6355 |  |
